# Human Amniotic Membrane Mesenchymal Stem Cells inhibit Neutrophil Extracellular Traps through TSG-6

**DOI:** 10.1038/s41598-017-10962-2

**Published:** 2017-09-29

**Authors:** Fátima Sofía Magaña-Guerrero, Alfredo Domínguez-López, Pamela Martínez-Aboytes, Beatriz Buentello-Volante, Yonathan Garfias

**Affiliations:** 1 Biología Celular y Tisular, Unidad de Investigación, Instituto de Oftalmología Fundación Conde de Valenciana, Chimalpopoca 14, Ciudad de México, 06800 Mexico; 2 Departamento de Bioquímica, Facultad de Medicina, Universidad Nacional Autónoma de México, Av. Universidad 3000, Ciudad de México, 04510 Mexico

## Abstract

The mesenchymal stem cells obtained from human amniotic membrane (hAMSC) possess immunosuppressive functions through soluble factors such as prostanoids and proteins; thus, they have been proposed to ameliorate inflammatory processes. On the other hand, activated neutrophils are cells of the first line of immune defense that are able to release extracellular traps (NETs). NETs are formed of DNA and granular components; however, the excessive release of NETs is associated with the development of autoimmune and chronic inflammatory diseases. In this study, we identified that conditioned medium (CM) from hAMSC was able to diminish NETs release, as well as the production of reactive oxygen species (ROS) and the mitochondrial membrane potential from LPS-stimulated mouse bone marrow-derived neutrophils (BMN). Interestingly, NETs inhibition, ROS levels decrease and mitochondrial membrane potential loss were reverted when LPS-stimulated murine derived BMN were exposed to the CM from hAMSC transfected with TSG-6-siRNA. Finally, rhTSG6 was able to significantly diminish NETs release in BMN. These data suggest an inhibition mechanism of NETs ROS-dependent in which TSG-6 participates. Consequently, we propose the hAMSC use as a therapeutic candidate in the treatment of inflammatory diseases in which NETs are involved.

## Introduction

Mesenchymal Stem Cells (MSC) are multipotent non-hematopoietic cells, which were first isolated from bone marrow^1,2^ and can be obtained from different adult and embryonic tissues such as liver, kidney, muscle, adipose and connective tissue, umbilical cord as well as term placenta^3,4^. In 2006, the International Society of Cell Therapy (ISCT) stated that the adhesion to plastic on *in vitro* culture, the ability to generate units-forming colony fibroblast (U-FCF), the expression of CD105, CD90, CD73, CD44, CD29 markers, the absence of hematopoietic markers CD45, CD19, CD34, CD11b, CD14 and CD79a expression as well as the potential of differentiation to one or more cell lineages are the minimal criteria for defining multipotent mesenchymal stromal cells^5^. Cumulative evidence has demonstrated that the MSC possess immunomodulatory activities and are able to regulate a wide range of immune cells. MSC inhibit the activation and function of dendritic cells^6,7^, decrease proliferation and effector functions of B and T lymphocytes^8–11^, and induce immunoregulatory responses on macrophages^12^. It has extensively been studied that MSC secrete a myriad of factors that are in charge of the immunoregulatory effects; among them, the TNF-α stimulated gene/protein 6 (TSG-6) is proposed as a key molecule in diminishing acute inflammation in different disease models such as corneal injury, peritonitis induced by yeast zymosan, bleomycin-induced lung injury and in preventing acute allogenic corneal rejection^13^. Additionally, induced-pluripotent stem cells (iPSC) are also capable of diminishing corneal inflammation in part by the expression of TSG-6^14^. The protein structure allows TSG-6 to interact with hyaluronic acid, chondroitin sulfate, inter-alpha-inhibitor (IαI), and is able to remodel extracellular matrix (ECM)^15^. TSG-6 is a 35-kDa protein that is secreted by a wide range of cell types in response to inflammatory mediators^16^. The gene expression of TSG-6 is tightly regulated and it is generally not constitutively expressed in adult mammalian tissues; however, there are exceptions to this, such as in human epidermis^17^, islets of the pancreas, adult central nervous system^18^, and the human amniotic membrane^19^. It has been described that TSG-6 secreted from bone marrow MSC possesses anti-inflammatory and immunomodulatory mechanisms in a murine traumatic brain injury^20^. More recently, it has been demonstrated the direct effect of secreted TSG-6 from human bone marrow mesenchymal stem cells by inhibiting the immune response through p38 and MEK mitogen-activated protein kinase pathway^21^.

Human amniotic membrane (hAM) is the inner layer of the placenta, which is in contact with the amniotic fluid and the fetus. The hAM consists of an epithelial layer, which is formed by cubical cells and an inner mesodermal tissue. It has been shown that hAM mesoderm is able to suppress the adaptive and innate immune responses^22–24^. In this context, there are cells that can be obtained from the hAM mesoderm; these are the human amniotic mesenchymal stromal cells (hAMSC). Similarly to MSC, hAMSC are able to differentiate into cells from each of the three germ layers and express pluripotency-associated stem cells markers^25^. Increasing evidence has demonstrated that hAMSC have immunosuppressive activities, inhibiting the innate and the adaptive immune systems. The hAMSC and their conditioned medium (CM) have the ability to inhibit T cells, reduce the expression of markers associated with Th1 and Th17 cell population, promote regulatory T cells and reduce the cytotoxicity of NK cells^26–28^. Also, hAMSC and their CM affect the differentiation of dendritic cells^29^. Moreover, hAMSC have the ability to participate in tissue reparation inhibiting fibrosis and promoting epithelialization. It has recently been shown that CM from hAMSC improved tissue regeneration in wound-healing models inducing the M1-to-M2 switch and enhancing M2-macrophages phenotype and their functions^30^. Furthermore, there are studies demonstrating that CM from AM-MSC have anti-inflammatory^31^, anti-neoplastic^32^ and immunomodulatory properties^33^. Therefore, there are soluble factors synthesized and secreted by hAMSC that are responsible in part of such activities. For example, prostaglandin E2 synthesized and secreted by hAMSC inhibit T cell proliferation^34^. Interestingly, TSG-6 presence has been evidenced in amniotic tissue as well as in cultured hAMSC^19^. However, as far as we know, the effect of hAMSC or their CM on the innate immune system, specifically on neutrophils, has not been sufficiently studied.

Neutrophils are cells that act as the first line of defense of the immune system^35^. Neutrophils activation has many consequences such as production of reactive oxygen species (ROS), lytic enzymes and antimicrobial peptides; also, they phagocytise and form and release neutrophil extracellular traps (NETs)^36^. NETs are structures made by DNA fibers and granular proteins like myeloperoxidase and elastase, antimicrobial peptides and citrullinated histones^37,38^. Though, it has been proposed that the formation of NETs is an immune mechanism to capture and eliminate pathogens and delimit the sites of inflammation^39,40^, there are still controversies on the effect of NETs release in health and disease phenomena. For example, NETs formation is beneficial in resolving the inflammatory reaction in gouty arthritis degrading IL-1β and IL-6 via serine proteases attached to NETs^41^; in contrast, NETs aggregation worsens the clinical symptoms in chronic pancreatitis inflammation through IL-17A, which is also contained in NETs^42^. Moreover, uncontrolled release of these traps and the exposure from their components has been associated to the development of some autoimmune diseases such as Systemic Lupus Erythematosus (SLE) and Rheumatoid Arthritis (RA)^43,44^. Additionally, endometriosis and chronic obstructive pulmonary disease pathogenesis have been associated to NETs formation^45,46^.

Despite therapies with hAMSC or their CM have been used to ameliorate acute and chronic inflammatory diseases, the precise mechanisms by which these cells or soluble factors exert their function is yet poorly understood. The aim of this study was to describe the role of TSG-6 produced by hAMSC on NETs release.

## Results

### Characterization of human Amniotic Mesenchymal Stem Cells

**Figure 1:**
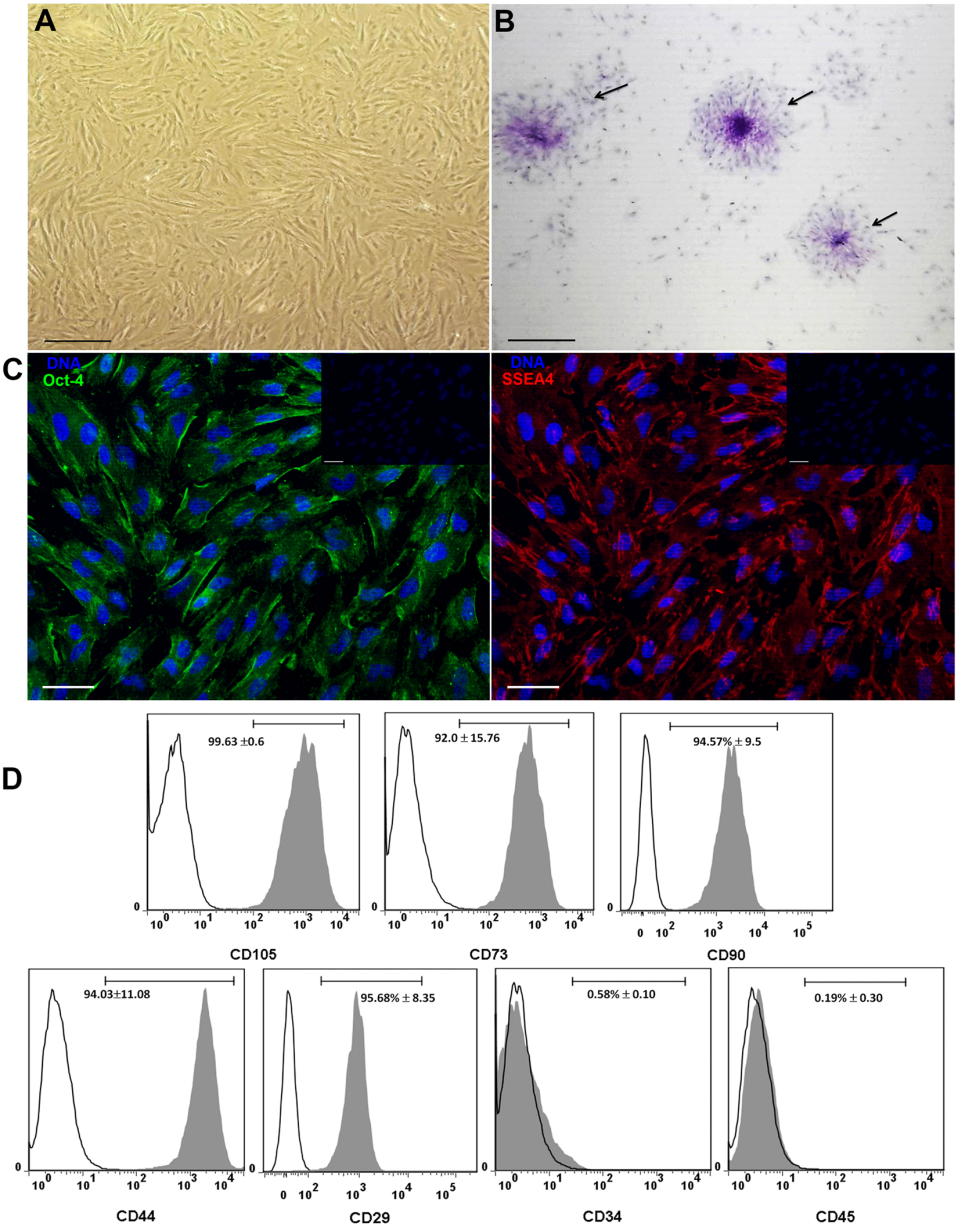
Cells obtained from human amniotic membrane mesoderm displayed mesenchymal stromal cells characteristics. Phase-contrast micrograph of hAMSC adhered to a polystyrene cell culture plate at 3^rd^ passage showing fibroblast morphology; the photograph was taken at 40x of magnification, scale bar 100 μm (**A**). The cells were cultured for ten days and stained with crystal violet, and a direct light micrograph was performed in order to identify the UFC (arrows); the photograph was magnified at 35x in a stereoscopic microscope, scale bar 100 μm (**B**). Fluorescence micrographs of hAMSC stained with pluripotent embryonic markers OCT-4 (left panel), and SSEA-4 (right panel). DAPI was used to identify their nuclei in both panels; scale bars represent 20 μm (**C**). hAMSC cells from 4^th^ passage were trypsinized and stained with antibodies against the indicated cell surface antigens and analyzed by flow cytometry. As shown, cells were positive to (>90%) CD105, CD73, CD90, CD44 and CD29; in contrast, they were negative to the expression of CD34 and CD45 hematopoietic-cells markers, inner numbers represent the mean ± SD (**D**). These are representative images from three different independent assays.

**Figure 2:**
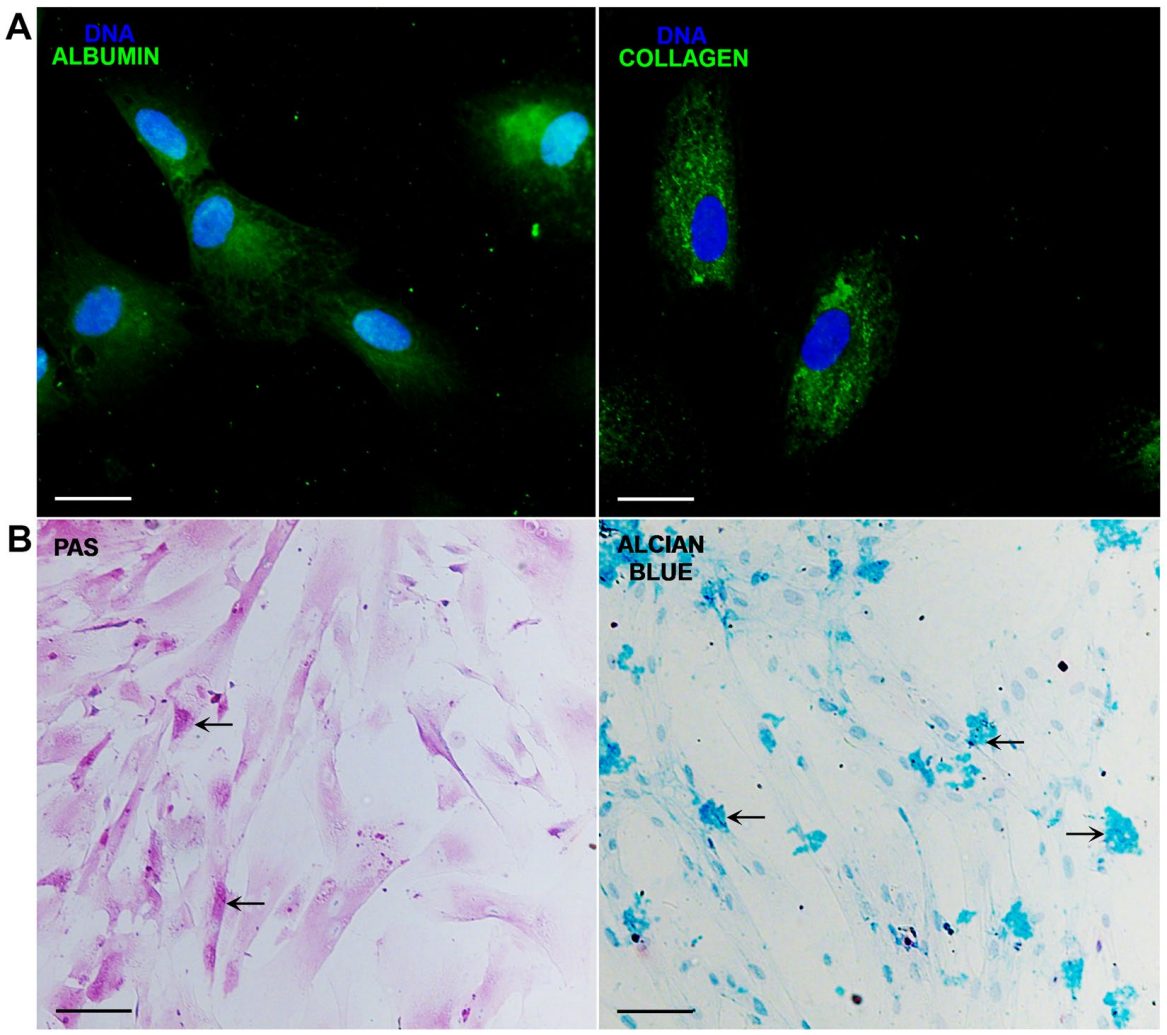
The hAMSC were able to transdifferentiate into hepatocyte-like and chondrocyte-like cells. hAMSC were cultured with medium either for hepatocytes or chondrocytes differentiation for 3 weeks, and immunostained for albumin or collagen, respectively. The differentiation was corroborated with a PAS and alcian blue stains for hepatocyte-like and chondrocyte-like cells, respectively. Fluorescence micrographs of hAMSC differentiated into hepatocyte-like cells expressing albumin (upper-left panel), and chondrocyte-like cells showing positivity to collagen-II (upper-right panel). Their nuclei were identified with DAPI. Scale bars represent 20 μm (**A**). Direct-light micrographs of hepatocytes-like cells producing glycogen demonstrated by PAS stain in their cytoplasm (lower-left panel), and of chondrocyte-like cells synthesizing proteoglycans and glycosaminoglycans stained with alcian blue (lower-right panel) (**B**). Scale bars represent 100 μm. These are representative images from three independent assays.

The cells obtained from the amnion were cultured in plastic flasks. And, according to the criteria proposed by the First International Workshop on Placenta Derived Stem Cells^47^, these cells attached to plastic adopted a fibroblastoid shape (Fig. 1A, upper-left panel); they made colony-forming units as observed with the crystal violet staining (Fig. 1B, upper-right panel). In addition to this, cells expressed embryonic/pluripotent stem cell markers such as Oct-4 and SSEA-4 proteins; both Oct-4 and SSEA-4 strongly stained the cytoplasm (Fig. 1C, lower-panel). When cell surface antigens from mesenchymal lineage were analyzed by flow cytometry, the cells expressed the MSC markers as follows: CD29: 95.68% ± 8.35; CD44: 94.03±11.08; CD73: 92.0 ± 15.76; CD90: 94.57% ± 9.5; CD105: 99.63 ±0.6, these results are expressed as mean ± SE; in contrast to these markers, the cells were negative (<1%) to CD45 and CD34 staining (Fig. 1D). In order to determine the ability of these cells to differentiate among other tissue lineages, they were cultured in the presence of two differentiation media for 3 weeks and immunofluorescence assays were performed. When the cells were cultured in the presence of the hepatogenic medium, the cells were positive to albumin and were able to synthesize glycogen, evaluated with PAS staining. On the other hand, in the presence of the chondrogenic medium, the cells were positive to collagen-II and were able to synthesize glycosaminoglycans evidenced with the alcian blue staining. Though, stem cells features were present in these cells (Fig. 2A and B).

### Conditioned medium from hAMSC inhibits NETs formation

**Figure 3:**
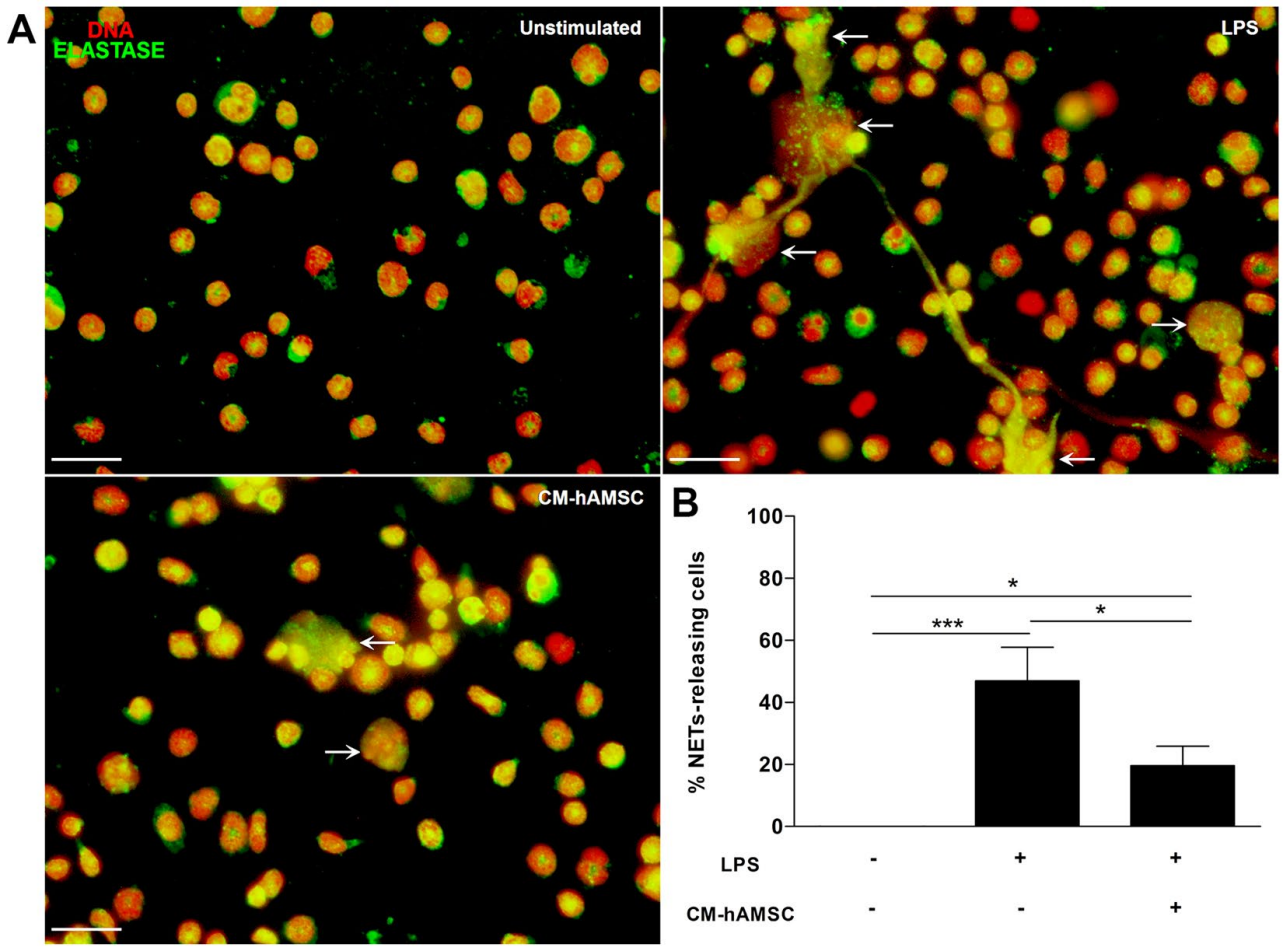
The soluble factors from hAMSC decrease the release of NETs. Murine neutrophils isolated from bone marrow were stimulated with LPS to induce the release of NETs and were incubated with CM from hAMSC. Fluorescence micrographs of unstimulated neutrophils (upper-left panel), LPS-stimulated neutrophils (upper-right panel, LPS) and LPS-neutrophils cultured with the CM from hAMSC (lower-left panel, CM-hAMSC). LPS-stimulated neutrophils liberated extracellular traps formed by elastase and DNA (white arrows). The neutrophils in contact with the soluble factors from hAMSC (CM-hAMSC) decrease the liberation of NETs. Scale bar represents 20 μm. These are representative images from three independent assays (**A**). Graphic represents the percentage of NETs releasing cells. The area of NETs was quantified with the Image J program from five random fields in each condition. Bars represent the mean percentage of NETs releasing cells ± SD (n = 3), *p < 0.05 (LPS vs. CM-hAMSC; unstimulated vs. CM-hAMSC); ***p < 0.001 (unstimulated vs. LPS) (**B**).

Mouse bone marrow isolated neutrophils were adhered to poly-*L*-lysine charged slides for 20 min. And afterwards, the medium was changed to either fresh medium without FBS or fresh CM derived from hAMSC as described in materials and methods, and the cells were exposed or not to LPS. The bone marrow murine neutrophils without stimulus released NETs in a basal manner; however, and as expected, LPS significantly increased (***p < 0.001) NETs release. Interestingly, there was a significant (*p < 0.05) inhibition of NETs release when the LPS-stimulated bone marrow murine neutrophils were exposed to fresh CM derived from hAMSC in comparison with that of NETs release by murine neutrophils exposed to LPS (Fig. 3).

### Human Amniotic Mesenchymal Stem Cells constitutively express TSG-6

**Figure 4:**
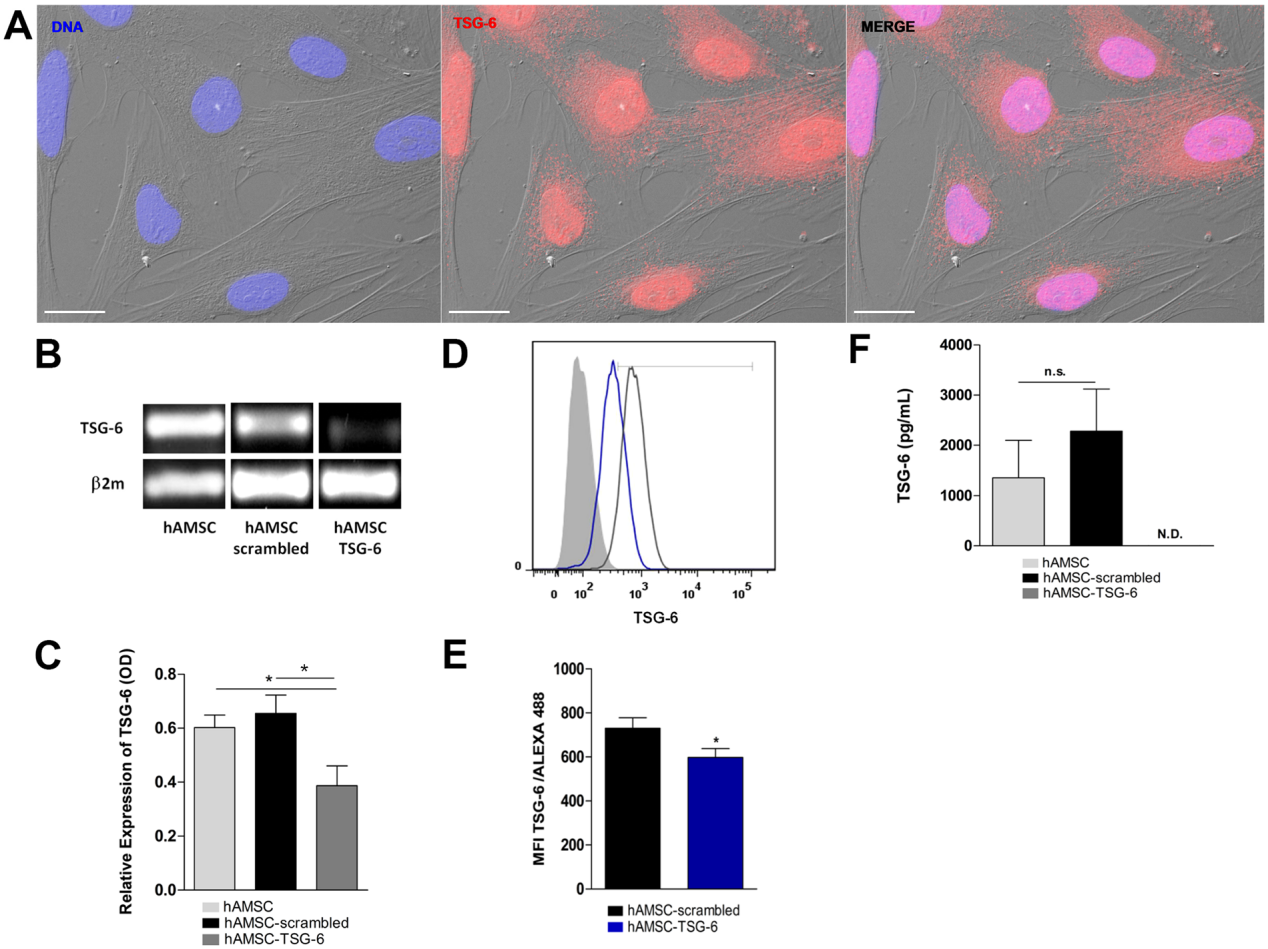
TNF-alpha Stimulated-Gene 6 protein (TSG-6) is expressed constitutively on hAMSC and is silenced by siRNA-TSG6 hAMSC were immunostained for TSG-6 together with DAPI for nuclei. Fluorescence micrograph of hAMSC stained for TSG-6 (middle-panel), the morphology of hAMSC was visualized with Differential Interference Contrast (DIC) microscopy. The merge immunostaining is shown in the image (right-panel). As shown in the panels, the TSG-6 protein is found in the nucleus and cytoplasm. Scale bar represents 20 μm. These images are representative from three independent experiments (**A**). The total RNA was extracted and PCR assays were performed on non-transfected hAMSC (hAMSC) and transfected cells with siRNA scrambled (hAMSC-scrambled) or siRNA TSG-6 (hAMSC-TSG-6). Image of PCR products on 1.5% agarose gel was revealed with ethidium bromide. The siRNA-scrambled was used to confirm the specific silencing of TSG-6. The expression of *tsg6* gene decreased on hAMSC-TSG-6 with respect to hAMSC-scrambled and hAMSC (**B**). Densitometry analyses were performed and data were normalized using β2 m as housekeeping. Data are expressed as mean ± SD (n = 3), *p < 0.05 (hAMSC vs. hAMSC-TSG-6; hAMSC-scrambled vs. hAMSC-TSG-6). Similar (n.s. = not statistically significant) *tsg-6* product level was found between hAMSC and hAMSC-scrambled (**C**). The transfected cells were immunostained for TSG-6 and the expression of the protein was analyzed by flow cytometry. Histogram in black represents the mean fluorescence intensity (MFI) of hAMSC-scrambled; histogram in blue represents the MIF of hAMSC-TSG-6 and gray histogram represents cells without staining (**D**). A decreased in the expression of TSG-6 was observed on hAMSC after silencing with siRNA TSG-6 respect to hAMSC-scrambled. Data are expressed as mean ± SD (n = 3), *p < 0.05 (hAMSC-scrambled vs. hAMSC-TSG-6) (**E**). The concentration of TSG-6 protein was analyzed in the CM of siRNA-transfected hAMSC by ELISA. Similar (n.s. = not statistically significant) TSG-6 levels were detectable in the CM of hAMSC and hAMSC-scrambled; however, undetectable levels (N.D.) of TSG-6 was shown in hAMSC-TSG-6. Data are expressed as mean ± SD (n = 3) (**F**).

As previously mentioned, TSG-6 is an immunosuppressive molecule constitutively expressed in epidermis, pancreas, adult central nervous system and interestingly amniotic membrane. Cumulative evidence shows that the primary function of TSG-6 is to protect the tissue from the damaging of inflammation and that many of the anti-inflammatory activities of MSC are mediated by TSG-6^20,21,48^. To corroborate that hAMSC have the ability to constitutively produce TSG-6, the cells were FBS-starved for 12 h before RNA extraction. After PCR reaction, the amplicon was resolved in 1.5% agarose gel, stained with ethidium iodide and observed in a transilluminator. The amplicon size was 178 pb as expected. In order to corroborate that the amplicon corresponded to *tsg*-6, automated DNA sequencing was performed, the nucleotide sequence of *tsg-6* amplicon disclosed a 100% homology with the wild type human *tsg-6* sequence. On the other hand, protein presence was determined by means of immunofluorescence. The TSG-6 protein was observed in the nucleus as well as in the cytoplasm; interestingly, the TSG-6 protein presented a diffuse distribution with a vesicular-like pattern in the nucleus as well as in the cytoplasm (Fig. 4).

### TSG-6 from hAMSC inhibits NETs formation

**Figure 5:**
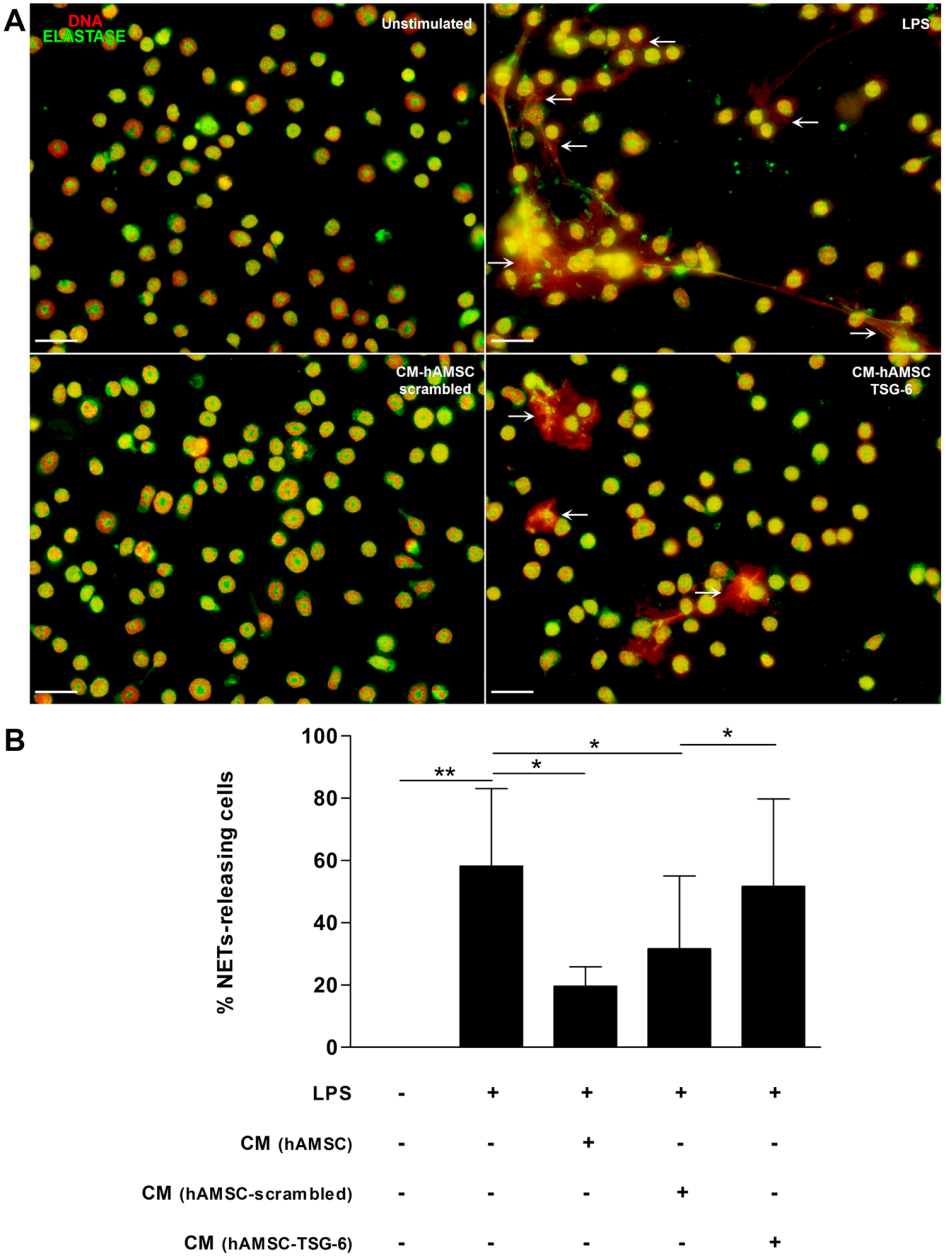
The conditioned medium (CM) from hAMSC decreases the release of NETs via TSG-6. Murine neutrophils were stimulated with LPS for NETs induction and incubated with the CM from hAMSC-scrambled or hAMSC-TSG-6. The siRNA-scrambled was used to corroborate that the specific silencing of TSG-6, did not affect the immunosuppressive effect of CM from hAMSC. After incubation, cells were stained for neutrophil elastase and the DNA was visualized with propidium iodide. Fluorescence micrographs of unstimulated neutrophils (upper-left panel), LPS-stimulated neutrophils (upper-right panel, LPS) and LPS-neutrophils cultured with the CM from hAMSC-scrambled (lower-left panel, CM-hAMSC-scrambled) or hAMSC-TSG-6 (lower-right panel, CM-hAMSC-TSG-6). The CM-hAMSC-scrambled decreases the release of NETs in the murine neutrophils (lower-left panel) respect the NETs in the LPS-stimulated neutrophils, while the CM-hAMSC-TSG-6 recovers the release of NETs in the neutrophils (lower-right panel). Scale bar represents 20μm (**A**). Graphic represents the percentage of NETs releasing cells. The area of NETs was quantified with the Image J program from five random fields in each condition. Bars represent the mean percentage of NETs releasing cells ± SD (n = 3), **p < 0.01 (unstimulated vs. LPS); *p < 0.05 (LPS vs. CM-hAMSC; LPS vs. CM-hAMSC-scrambled; CM-hAMSC-scrambled vs. CM-hAMSC-TSG-6); n.s. = not statistically significant (CM-hAMSC vs. CM- hAMSC-scrambled) (**B**).

In order to identify the role of TSG-6 in NETs formation, gene knockdown of TSG-6 was performed on hAMSC using a specific siRNA. Cells transfected with siRNA specific to TSG-6 significantly (*p < 0.05) reduced the expression of its transcript compared to that of the scrambled control siRNA transfected cells that showed no differences among the non-transfected cells; similarly, TSG-6 siRNA transfection inhibited significantly (*p < 0.05) the TSG-6 protein presence in comparison to that of the scrambled control siRNA and non-transfected cells. Finally, and in order to confirm TSG-6 inhibition affected its presence in the CM, ELISA was performed and undetectable levels of TSG-6 were found (Fig. 4). Once the hAMSC cells were transfected with TSG-6 and scrambled siRNA, their respective supernatants were used for the following assays. Mouse bone marrow neutrophils stimulated or not with LPS were exposed to different conditioned media: hAMSC-conditioned medium (CM-hAMSC), conditioned medium from hAMSC transfected with TSG-6-siRNA (CM-hAMSC-TSG-6), or conditioned medium from hAMSC transfected with scrambled siRNA (CM-hAMSC-scrambled). After adhering the murine neutrophils to poly-*L*-lysine charged slides for 20 min, the cells were stimulated or not with LPS and exposed to the different conditioned media (afore mentioned) for additional 90 min. The cells were fixed and NETs quantification was performed. Murine bone marrow isolated neutrophils cultured in the presence of CM of non-transfected hAMSC released low levels of NETs when exposed to LPS in comparison those with the LPS-stimulated neutrophils in the presence of fresh medium (*p < 0.05). Interestingly, when neutrophils were exposed to LPS in the presence of CM-hAMSC-TSG-6 there was a significant (*p < 0.05) increase of NETs levels in comparison with that of the NETs levels released by murine neutrophils-LPS stimulated in the presence of CM of hAMSC-scrambled as well as with the CM derived from non-transfected hAMSC (*p < 0.05).Contrariwise, there was no difference between the NETs levels of the neutrophils exposed to LPS in the presence of CM-hAMSC-TSG-6 and the NETs levels of the LPS-exposed neutrophils without CM (Fig. 5).

### rhTSG-6 reduces NETS formation

**Figure 6:**
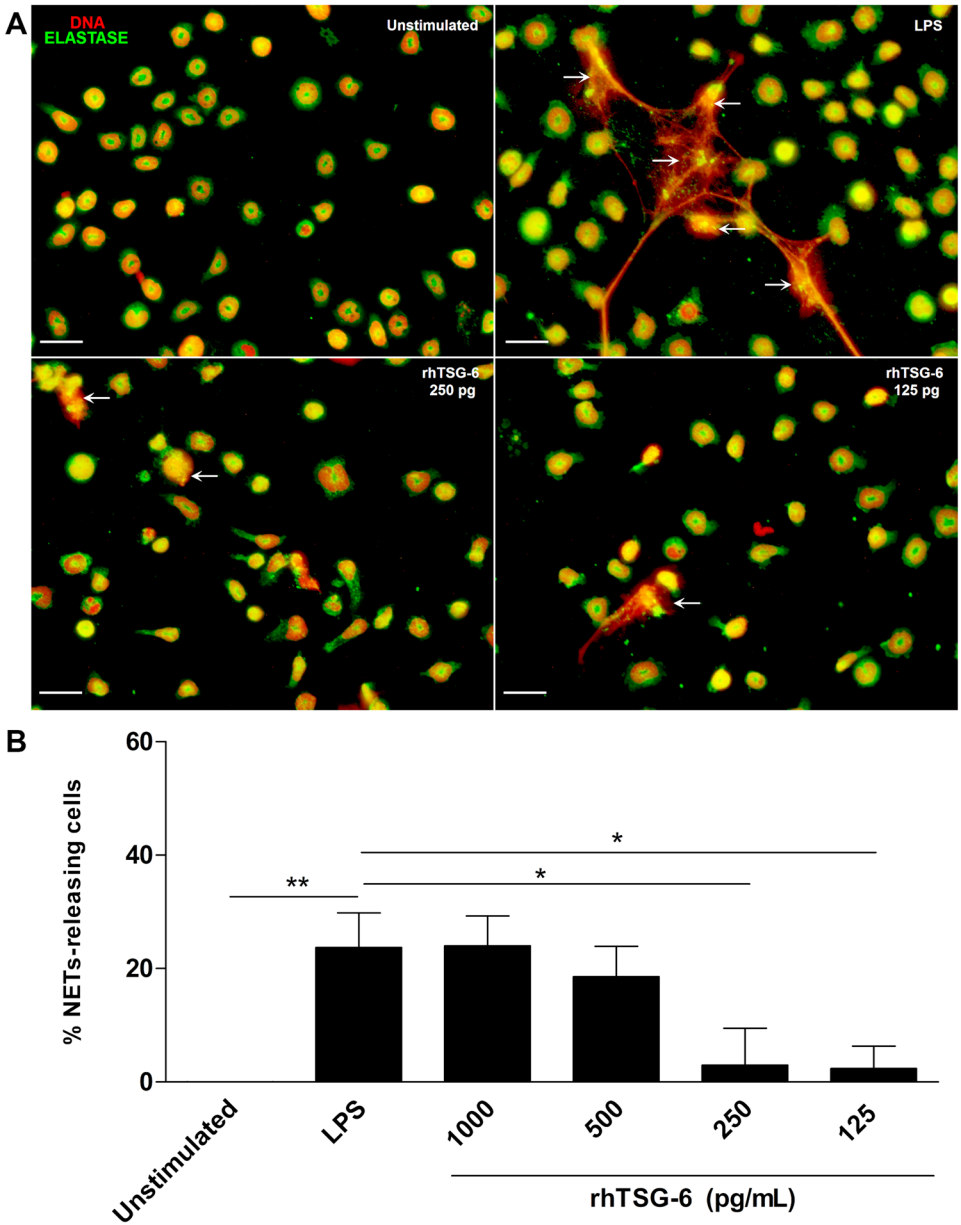
Recombinant human TSG-6 decreases the release of NETs. Murine neutrophils were stimulated with LPS for NETs induction and incubated with serial dilutions of rhTSG-6. After incubation, cells were stained for neutrophil elastase and the DNA was visualized with propidium iodide. These data are representative of three independent experiments (**A**). Graphic of the percentage of NETs releasing cells from LPS-stimulated neutrophils incubated with different doses of rhTSG-6. The rhTSG-6 decreases the release in a dose-dependent manner. The area of NETs was quantified with the Image J program from five random fields in each condition. Bars represent the mean percentage of NETs releasing cells ± SD (n = 3), **p < 0.01 (unstimulated vs. LPS), *p < 0.05 (LPS vs. rhTSG6 250 pg/ml; LPS vs. rhTSG6 125 pg/ml) (**B**).

In order to directly determine the effect of TSG-6 on NETs release by LPS-stimulated mouse bone marrow neutrophils, different doses of rhTSG-6 were used; interestingly, 250 and 125 pg/ml of rhTSG-6 showed a decrease with significant differences (*p < 0.05) in comparison to those cells stimulated with LPS and cultured with medium without rhTSG-6 (Fig. 6).

### NETs inhibition through TSG-6 is ROS dependent

**Figure 7:**
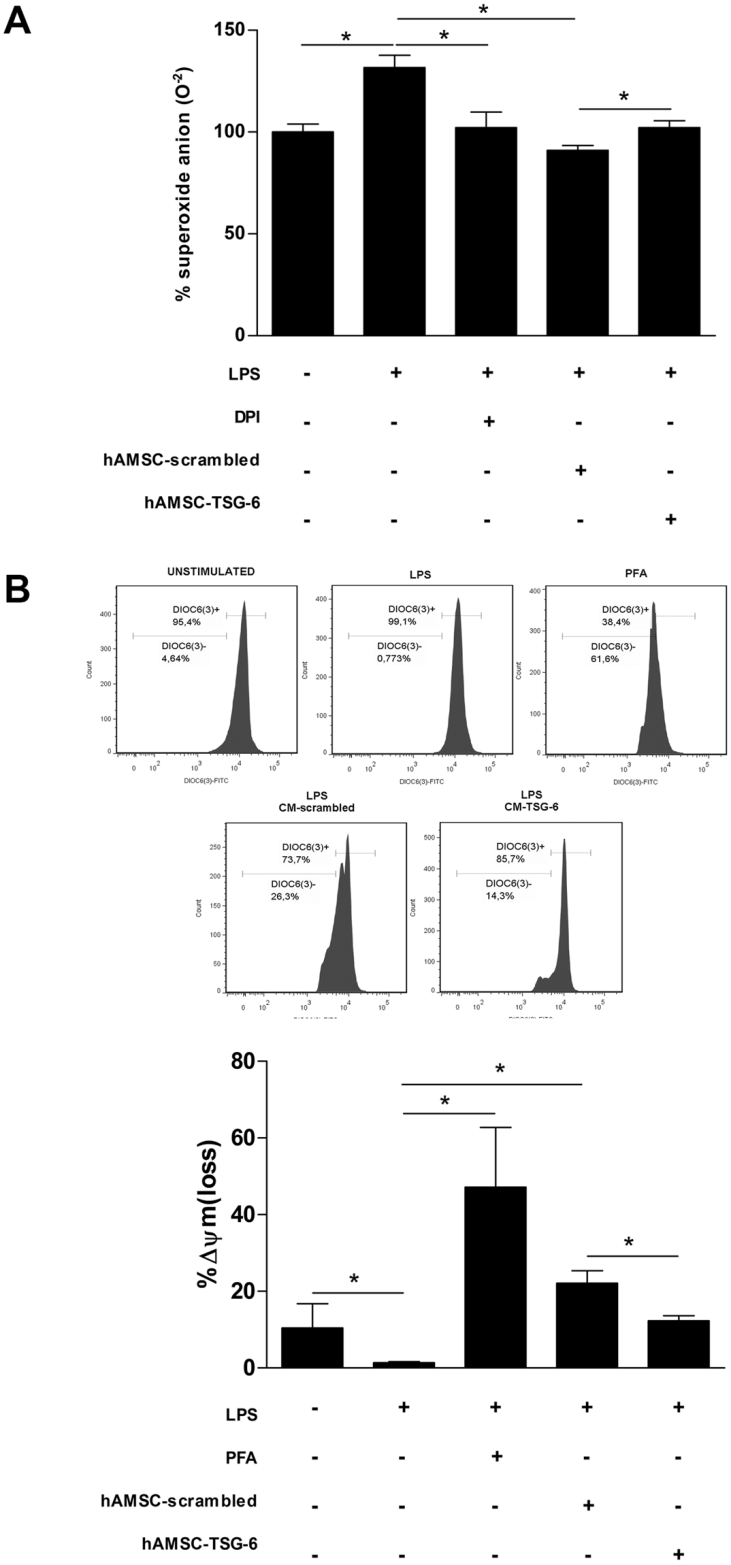
The decrease of NETs release through TSG-6 is ROS dependent. Murine neutrophils were stimulated with LPS and incubated with the CM-hAMSC-scrambled or CM-hAMSC-TSG-6 during 30 min. The siRNA-scrambled was used to corroborate that the specific silencing of TSG-6, did not affect the immunosuppressive effect of CM from hAMSC. The ROS production was determined with the NBT reduction assay. Data are expressed as the percentage of superoxide anion production. Graphic of percentage of superoxide anion production in LPS-stimulated neutrophils cultured with the CM-hAMSC scrambled or CM-hAMSC-TSG-6. Bars represent the mean percentage of superoxide anion ± SD (n = 3), *p < 0.05 (unstimulated vs. LPS; LPS vs. DPI; LPS vs. CM-hAMSC-scrambled; CM-hAMSC-scrambled vs. CM-hAMSC-TSG-6). DPI (Diphenyleneiodonium) an inhibitor of NADPH complex was used as inhibitor of ROS and superoxide anion production (**A**). The mitochondrial membrane potential was measured with the fluorescent dye DiOC6(3) and analyzed by flow cytometry. Data are expressed as the percentage of Δψ_m_ loss. Graphic of the percentage of Δψ_m_ loss of LPS-stimulated neutrophils cultured with the CM-hAMSC-scrambled or CM-hAMSC-TSG-6. The percentage of Δψ_m_ loss of LPS-stimulated neutrophils cultured with the CM-hAMSC-scrambled increased with respect to LPS-stimulated neutrophils only with fresh medium or cultured in the presence of CM-hAMSC-TSG-6. *p*-formaldehyde (PFA) treated LPS-stimulated neutrophils were used as a positive control of the Δψ_m_ loss. Bars represent the mean percentage of Δψ_m_ loss ± SD (n = 3), *p < 0.05 (unstimulated vs. LPS; LPS vs. PFA; LPS vs. CM-hAMSC-scrambled; CM-hAMSC-scrambled vs. CM-hAMSC-TSG-6 (**B**).

The inhibition of the oxidase NADPH complex significantly reduces NETs release^49^. In order to determine whether the inhibition of NETs by CM of hAMSC was associated with a reduction on the activity of oxidase NAPDH, NBT assays were performed. Bone marrow isolated neutrophils in the presence of fresh medium without stimulus showed a basal activity, which was considered as the 100% of the ROS production. As expected, the levels of ROS production significantly (*p < 0.05) increased 30% from the basal ROS production when the cells were cultured in the presence of fresh medium with LPS. Interestingly, the CM-hAMSC-scrambled significantly (*p < 0.05) inhibited up to 25% of the ROS production by LPS-stimulated murine bone marrow neutrophils in comparison to that of the ROS production from LPS-stimulated neutrophils only with fresh medium; this inhibition on ROS production was similar to that of the LPS-stimulated neutrophils ROS production using the NADPH inhibitor Diphenyleneiodonium (DPI)^50^. When the LPS-stimulated neutrophils were exposed to the CM-hAMSC-TSG-6, they significantly (*p < 0.05) increased the ROS production respect with the cells cultured with the CM-hAMSC-scrambled (Fig. 7).

### Neutrophils mitochondrial membrane potential Δψ_m_ loss is affected by TSG-6

The mitochondrial membrane potential is an indicator for the energetic state of mitochondria and their correct function. The proper mitochondrial functioning is associated to the maintenance of the metabolic activity and the electron transport that permits the production and release of ATP^51^. Neutrophils require significant energy expenditure for the development of the various effector mechanisms they perform. It has been reported that alterations in mitochondrial function cause a decrease in the membrane potential (Δψ_m_), affecting the metabolic activity and causing a decrease in the amount of ATP, with the consequent low production capacity of ROS. The alteration of the mitochondrial processes results in an altered phagocytic capacity and chemotaxis of the neutrophils^52,53^. When bone marrow isolated neutrophils were exposed to LPS, there was a slight increase of the Δψ_m_ loss. However, there was an increase of the Δψ_m_ loss in a similar way of *p*-formaldehyde treated LPS-stimulated neutrophils used as a positive control of the Δψ_m_ loss, when the LPS-stimulated neutrophils were exposed to the CM of the hAMSC-scrambled. This loss of the Δψ_m_ was significantly (*p < 0.05) reduced when the LPS-stimulated neutrophils were incubated with the CM-hAMSC-TSG-6  (Fig. 7).

## Discussion

In the present study, the cells obtained from the mesoderm of human amnion (hAMSC) showed stem cells characteristics according to the ISCT and their CM inhibited LPS-stimulated murine bone marrow neutrophils NETs release. NETs release inhibition was reverted when the LPS-stimulated murine neutrophils were exposed to CM from TSG-6-silenced hAMSC. Similarly, CM from hAMSC were able to decrease the production of ROS in LPS-stimulated murine neutrophils, and this inhibition was regressed when the LPS-stimulated neutrophils were cultured in the presence of TSG-6 silenced hAMSC. Furthermore, CM of hAMSC augmented the LPS-stimulated Δψ_m_ loss, and the CM from TSG-6-silenced hAMSC reverted this augmentation. Finally, NETs release was inhibited in the presence of rhTSG-6 in a dose dependent manner in order to corroborate the direct effect of TSG-6 on the immune innate response.

The NETs release is an effector function from neutrophils described more than 10 years ago^36^; at that time, the main function attributed of these traps were their antimicrobial activity. However, it has currently been shown that excessive or uncontrolled NETs release is associated with multiple autoimmune and chronic inflammatory diseases. The generation of inflammation on NETs release is associated to its high content of degrading enzymes and oxidizing agents causing tissue damage. Identifying new mechanisms to reduce NETs release is essential to diminish this proinflammatory pathway. Recently, the use of mesenchymal cells as a therapy to ameliorate inflammatory processes has been increasing^13^. The cells we isolated from human amniotic membrane (hAMSC) presented mesenchymal stromal cells features according to the ISCT. hAMSC were positive (>90%) to the expression of defined cell surface markers such as CD90, CD29, CD73, CD105 and CD44, while negative to the expression of both hematopoietic markers CD34 and CD45; these results are in accordance to those previously reported^47^. Other features such as the expression of early stem cells markers e.g. SSEA-4 and Oct-4, colony forming units and hepatic, and chondroblasts differentiation support that these are stem cells^54^. The rationale for using CM in the present study is due to the fact that MSC are able to synthesize and secrete anti-inflammatory molecules. When NETs release was diminished in LPS-stimulated bone marrow neutrophils cultured in the presence of CM from hAMSC, we hypothesized that soluble molecules were in part responsible of such anti-inflammatory effect. As stated before, TSG-6 has been proposed as a key molecule in diminishing acute inflammation by MSC^20,21,48^. According with the results presented by Zhang *et al*., we found that hAMSC constitutively express TSG-6 at both transcriptional and protein levels^19^; however, in the present study, *tsg-6* amplicon identity was corroborated by automatic DNA sequencing which had 100% homology to the human *tsg-6-*gen. In order to determine the effect of TSG-6 protein in NETs release, knockdown of *tsg-6* by siRNA was performed. Although the results of the siRNA knock-down of TSG-6 is only partial, shown by means of RT-PCR and flow cytometry, there were undetectable levels of TSG-6 measured by ELISA, suggesting the absence of TSG-6 protein in the CM. Interestingly, the LPS-stimulated neutrophils reverted the inhibition of the release of NETs when were cultured in the presence of hAMSC silenced to TSG-6 expression, suggesting that TSG-6 is, in part, responsible for NETs release inhibition. In a more direct manner, NETs release was also significantly diminished when the LPS-stimulated neutrophils were cultured in the presence of rhTSG-6 compared to that of LPS-stimulated neutrophils without rhTSG-6. Unexpectedly, NETs release inhibition was only achieved at low concentrations of rhTSG-6, while higher rhTSG-6 concentrations failed to reduce NETs formation, in this context, it has been reported that rhTSG-6 tends to self-aggregate which could explain this effect in our model^55^.

The TSG-6 protein forms complexes with inter-alpha-inhibitor (IαI) and these complexes formation inhibits the enzymatic activity of elastase; in this context, the enzymatic activity of elastase and myeloperoxidase is essential for NETs release^56–58^. The role of TSG-6 in inhibiting NETs release down regulating the enzymatic activities of elastase and myeloperoxidase in our model cannot be ruled out.

Activation of NADPH oxidase and consequently, ROS generation, are essential mechanisms for NETs formation and release^59,60^. Therefore, we determined ROS production in LPS-stimulated neutrophils in the presence of CM from hAMSC transfected either with siRNA to TSG-6 or scrambled siRNA to in turn determine the effect of TSG-6 on ROS production; we found a decrease in superoxide anion concentration in the LPS-stimulated neutrophils that were cultured in the presence of the CM from scrambled-hAMSC. Meanwhile, this decrease was recovered when the LPS-stimulated neutrophils were incubated in the presence of CM from hAMSC-TSG-6. Diphenyleneiodonium (DPI) was used as a positive control for NADPH oxidase inhibition, suggesting that TSG-6 from hAMSC is participating in the activation of this central enzyme on NETs generation. These results are in accordance to those reported by Khan, *et al*., who described that MSC derived from Wharton’s jelly were able to decrease the NADPH oxidase gene expression in adult and neonatal neutrophils^61^.

With respect to the effect of TSG-6 on the mitochondrial function, we found modifications in the mitochondrial potential with an increase in the percentage in the Δψ_m_ in the neutrophils cultured with the CM-hAMSC-scrambled. This change in the mitochondrial potential could be a cause for a less production of superoxide anion found in the previous results. Whereas, when the neutrophils were incubated in the presence of CM from hAMSC with TSG-6 silenced, we observed a decrease in the percentage of Δψ_m_ on the cells, together with the recovered capacity to generate ROS. These results suggest that the CM of hAMSC could affect the mitochondrial function in a TSG-6-dependent form. Our findings agree with what Hu J. *et al*. reported, that bone marrow mesenchymal stem cells (BMSC) are able to attenuate interleukin-1β-induced inflammation by the inhibition of NF-κB signaling and the mitochondrial pathway on lumbar disc cells^62^.

In conclusion, our study revealed that, the hAMSC releases several soluble factors with anti-inflammatory capacity. One of these factors is the TSG-6 protein, which, according to our results, participates in the inhibition of the release of NET by a mechanism that involves the modulation of mitochondrial function and is ROS-dependent inhibition. However, the events that are carried out in this mechanism of inhibition are still unknown; so, further studies are needed.

## Materials and Methods

### Reagents

Trypsin 10X; Collagenase II; Fetal Bovine Serum (FBS); Dispase II; Phosphate Buffer Solution (PBS, pH 7.2), Opti-MEM Reduced Serum Medium, Penicillin (10,000 IU/ml)/Streptomycin (10 mg/ml) (PS), Lipofectamine 2000 were obtained from Invitrogen (Waltham, MA). Dulbecco’s Modified Eagle Medium/Nutrient Mixture/F-12 (DMEM/F-12), Trypan blue, *p*-formaldehyde, poly-*L*-lysine, Triton 100X, dimethylsulfoxide (DMSO), Roswell Park Memorial Institute (RPMI) medium-1640; Lipopolysaccharide (LPS) from *E*. *coli* strain *OB111:B4;* Nitroblue Tetrazolium (NBT), Propidium iodide (PI), human TSG-6 ELISA kit, were purchased from Sigma-Aldrich (Saint Louis, MI). Ethylenediaminetetraacetic acid (EDTA) was obtained from Promega (Madison, WI). PerCPCy5.7-conjugated anti-CD44, PE-conjugated anti-CD34, FITC-conjugated anti-CD29, APC-conjugated anti-CD105, PE-conjugated-CD73 and PerCP-Cy5.5-conjugated anti-CD45 were obtained from e-Bioscience (San Diego, CA). Purified antibodies: anti-elastase, anti-albumin, anti-collagen II, anti-TSG-6, anti-SSEA-4 and anti-Oct-4 were purchased from Abcam (Cambridge, England). AlexaFluor-488 and AlexaFluor-594 conjugated goat anti-rabbit antibodies were obtained from LifeTechnologies (Eugene, OR). Bovine Serum Albumin (BSA) was obtained from Calbiochem (San Diego, CA). Cell culture flasks, 24 wells plastic plates and 96-wells plastic plates were purchased from Corning Inc (Corning, NY). RNeasy and Omniscript kits were obtained from Qiagen (Hilden, Germany). Vectashield- 4′,6-diamidino-2-phenylindole (DAPI) was purchased from (Vector Laboratories (San Diego, CA); Neutrophils Isolation Kit mouse was obtained from Miltenyi Biotec (Bergisch Gladbach, Germany). Small interfering RNA (siRNA) TSG-6 and the scrambled control siRNA (Santa Cruz, TX). KAPA2G Fast Ready Mix PCR Kit was purchased from KapaBiosystems (Boston, MA). Recombinant human TSG-6 (rhTSG-6) was purchased from R&D systems (Minneapolis, MN). 3,3′-Dihexyloxacarbocyanine Iodide (DiOC6(3)) from molecular probes (Eugene, OR).

### Ethical Statements

All the methods and experimental protocols were carried out in accordance to the Ethical Review Board (ERB) from the Institute of Ophthalmology Conde de Valenciana. The protocol was approved by the ERB from the Institute of Ophthalmology Conde de Valenciana. The informed consent was obtained from women who voluntary donated their placentas.

### Isolation of Human Amniotic Mesenchymal Stromal Cells (hAMSC)

Human term placentas were processed immediately after delivery. The informed consent was obtained from women who voluntary donated their placentas. Following the similar the criteria for donation reported by Chávez-García, *et al*., a total of five (18–37 years old) donors were included in this study^63^.

The amnion was separated mechanically from the chorion, which was discarded, while the amnion was rinsed with PBS. Afterwards, the amnion was cut on small pieces and incubated with 0.25% trypsin/EDTA (0.05%) for 10 min at 37 °C, 5% CO_2_ with gentle shaking. The pieces of amnion were rinsed with PBS and the amniotic epithelium was removed by enzymatic digestion using 0.25% trypsin/EDTA (0.05%) during 40 min with gentle shaking every 10 min; then, the fragments of amnion were chopped and digested with 0.75 mg/ml of collagenase II during 90 min at 37 °C, 5% CO_2_ atmosphere with gentle shaking every 10 min. After passing the suspension on a nylon mesh, the cells were centrifuged at 800 x *g* for 5 min at 10 °C. The supernatant was discarded and the human amniotic mesenchymal stem cells (hAMSC) were cultured in DMEM/F12 medium supplemented with 20% of FBS and 1% of penicillin/streptomycin. The medium was replaced every other day. Passages 2–4 of hAMSC were used to perform all the experimental procedures, unless otherwise stated. The hAMSC cell characterization and histological stains were performed in all the cells obtained from all the donors. Transcript identification and immunofluorescence for TSG-6 was performed in three different donors. For the CM preparation, cells from three different donors were used, and replicates from each donor were performed, unless otherwise stated.

### Characterization of hAMSC

In accordance with the First International Workshop on Placenta Derived Stem Cells^47^, we used the minimal criteria for defining hAMSC: (1) Adherence to plastic and formation of fibroblast colony-forming unit; (2) Identification of a specific pattern of cell surface antigen expression; and, (3) Differentiation towards one or more lineages. ***Colony-forming unit assay***: in order to perform this assay, the hAMSC were seeded at two cells per cm^2^ and cultured for ten days in a plastic cell culture bottle. After this period, cells were stained with 0.5% crystal violet in methanol for 5 min and rinsed with deionized water. Colonies formed were considered when > 2 mm in diameter. ***Phenotypic characterization of hAMSC identifying cell surface antigens expression by flow cytometry***: briefly, cells were detached from the plastic cell culture bottles using 0.25% trypsin/EDTA (0.05%) for 5 min at 37 °C, complete medium (DMEM/F12, supplemented with 10% FBS and 1% of PS) was added to avoid the trypsin enzymatic reaction; cells were rinsed twice with PBS. Cells were incubated with different fluorochrome-conjugated antibodies for 30 min in the darkness. After incubation time, cells were rinsed twice with PBS and 1 × 10^4^ cells were acquired in a FACS Verse flow cytometer (BD, San Diego, CA); the analyses were performed using FlowJo 7.6 software (FlowJo LLC, Ashland, OR, USA). Identification of early markers of differentiation was performed by means of immunofluorescence with antibodies to SSEA-4 and Oct-4 as described below. ***In vitro differentiation assays of hAMSC***: hAMSC (2 × 10^3^) were cultured on poly-*L*-lysine charged slides on a 24-plastic-wells plate with complete medium. At day three, the medium was replaced with differentiation medium and cells were maintained for at least three weeks; the differentiation medium was replaced every other day. All differentiation media were composed of DMEM/F12 supplemented with 10% FBS and 1% PS with the specific differentiation factors: for chondrogenesis (6.25 μg/ml insulin, 10 μg/L rhTGF-β1, 300 nmol/L ascorbic acid) and for hepatogenesis (100 nmol/L dexamethasone and 100 nmol/L insulin)^64^. After differentiation assays, and in order to corroborate the cell linage differentiation, immunofluorescence assays and specific histological stains were performed as indicated below.

### Histological stains

In order to corroborate the differentiation capacity of hAMSC to chondrocytes, alcian blue staining was performed to determine the glycosaminoglycans. To corroborate the hepatic differentiation, Periodic Acid-Schiff staining (PAS) was performed to identify glycogen, glycoproteins and glycolipids.

### Immunofluorescence assays

All immunofluorescence assays were performed as follows with modifications depending on each experiment: the cells on poly-*L*-lysine-coated glass cover slips were fixed with 4% *p*-formaldehyde for 10 min at 4 °C and incubated with blocking buffer (5%-BSA, 0.1% Triton 100X and 0.1 M PBS) for 2 h at room temperature (RT). Subsequently, the purified primary antibody was added and incubated overnight at 4 °C; the excess of antibody was rinsed once with PBS-0.05% tween (PBS-T). Afterwards, the samples were incubated 2 h either with AlexaFluor-594-conjugated or AlexaFluor-488 goat anti-rabbit antibodies at RT. And finally, the specimens were rinsed twice with PBS-T and mounted with Vectashield-DAPI. The images were acquired with an ApoTome II microscope, and analyzed using Axiovison 2.0 software (Carl Zeiss, Jena, Germany).

### Preparation of conditioned medium (CM)

The hAMSC either with or without siRNA treatment (see below), were plated in 6-well plates at a density of 1.5 × 10^5^ cells in a final volume of 2 ml per well of DMEM-F12 supplemented medium. Twenty-four hours after the hAMSC were adhered the supernatant was replaced with fresh DMEM-F12 supplemented medium or DMEM-F12 alone in the case of the siRNA transfection (see below). The cells were cultured for 24 h at 37 °C and 5% CO_2_ atmosphere. After this culture time the medium was collected and was considered as conditioned medium (CM). All the conditioned media were used for the assays fresh, immediately after collection. For the NET-inhibition experiments the neutrophils were incubated with CM at 100% together with the LPS-stimulus.

### Identification of transcript and protein of TSG-6 on hAMSC

Expression and presence of TSG-6 were performed by RT-PCR and immunofluorescence, respectively. In order to determine whether hAMSC constitutively expressed *tsg-6*, ***RT-PCR assays*** were performed using 1.5 × 10^5^ hAMSC. Total RNA was obtained using the RNeasy Mini Kit following the manufacturer’s instructions. Retrotranscription assays were performed using 100 ng of total RNA with the Omniscript kit according to the manufacturer’s instructions. The PCR assays were performed with the KAPA2G Fast Ready Mix PCR Kit in accordance with the manufacturer’s instructions using the following primers: *tsg-6* forward: 5′-GTCTGTGCTGCTGGATGGAT-3′, *tsg-6* reverse: 5′-TAAAGACGCCACCACACTCC-3′; *β2 m f*orward: 5′-CACCCCCACTGAAAAAGATG-3′, *β2 m* reverse 5′-ATATTAAAAAGCAAGCAAGCA-3′. The PCR products were visualized in 1.5% agarose gels stained with ethidium bromide, the images were captured and digitalized using G-Box system and Gene Snap Software version 7.12.6 (Syngene, London, UK). The mRNA expression was calculated by densitometry analysis after normalization with respect to the housekeeping beta-2 microglobullin (*β2 m)*. On the other hand, to determine the presence of TSG-6 protein, ***immunofluorescence assays*** were performed as follows: hAMSC (3 × 10^3^) were seeded on poly-L-lysine-coated glass cover slips in a 24-flat wells plate. After attachment to the cover slips, cells were fixed, blocked and immunofluorescence assays using anti-TSG6 as the primary antibody were performed as described before. ***Automated DNA sequencing***: *tsg-6* products amplified by RT-PCR were directly sequenced to confirm the identity of the amplicon. Nucleotide sequence was achieved using the BigDye terminator kit (Applied Biosystems, Foster City, CA) of fluorescently labeled terminators and analyzing the samples in an ABI 310 DNA Sequencer (Applied Biosystems). Sequences were compared to the published human wild type *tsg-6* gene (Ensemble transcript ID number ENSG00000123610).

### Transfection of hAMSC with TSG-6 siRNA

In order to knockdown the expression of *tsg-6*, hAMSC were transfected with *tsg-6* siRNA. Cells were seeded at a density of 1.5 × 10^5^ on a 6-wells plate with complete culture medium. The complete medium was replaced with medium without FBS and antibiotics 16 h before transfection. The transfection was performed in the presence of Opti-MEM and Lipofectamine 2000. The siRNA of *tsg-6* and the scrambled control siRNA (Santa Cruz, TX, USA; sc-39819; sc-37007, respectively) were diluted with Opti-MEM at a concentration of 20 pM, according to the manufacturer’s instructions and mixed with Lipofectamine 2000 to form the liposomes for 20 min at RT. The liposomes were added to hAMSC with 1 ml of Opti-MEM, and cells were incubated for 6 h. After transfection, the medium was replaced with 2 ml of complete fresh medium for an additional period time of 24 h. The cells were cultured at 37 °C and 5% CO_2_ atmosphere. At the end of the culture, the supernatants were obtained and immediately used as CM. To confirm knockdown of *tsg-6*, transcript and protein presence were assessed by means of RT-PCR and flow cytometry, as described before^65^.

### Enzyme Linked Immuno Sorbent Assay to TSG-6

TSG-6 protein levels in the conditioned media from non-transfected hAMSC (CM), scrambled-control siRNA-transfected hAMSC (hAMSC-scrambled) and from TSG-6-transfected hAMSC (hAMS-TSG-6) were determined by ELISA. Twenty-four hours after hAMSC transfection with their respective siRNAs, the CM were collected and analyzed by ELISA using the human TSG-6 ELISA kit according the manufacturer’s instructions.

### Isolation of bone marrow murine neutrophils

Bone marrow cells were obtained from 6–8 week-old BALB/c male mice as previously reported with modifications^66^. Briefly, tibias and femurs were obtained and dissected after mice euthanasia. The bone marrow cells were flushed with murine neutrophils buffer (1% glucose and 0.5% BSA in Hank’s Balanced Salt Solution) using a syringe with 27-G needle. The pooled bone marrow was suspended by gentle pipetting followed by filtration thorough a 100-μm-nylon cell strainer to remove cell clumps and bone particles. The suspension was centrifuged at 300x *g* for 10 min at 10 °C. The neutrophils were purified with a Neutrophils Isolation Kit according to the manufacturer’s instructions. Neutrophils viability was performed using the trypan blue exclusion method and the cell purity was corroborated determining CD11b and elastase by flow cytometry as described above.

### NETs induction and quantitation

NETs induction and quantification were performed as previously described with modifications^67^. Briefly, mouse neutrophils (4 × 10^4^) were seeded on 13 mm poly-*L*-lysine charged glass coverslips in 24-well-plates in RPMI. The plates were incubated for 20 min at 37 °C to allow adhesion of the cells, and subsequently, the cells were exposed or not to LPS; afterwards, cells were incubated for 90 min. LPS concentration was standardized as well as the time in which optimal NETs formation was observed without any NETs degradation (32 μg/ml and 90 min, respectively). After the final incubation, cells were fixed with *p*-formaldehyde for 10 min at RT. The coverslips with the cells were rinsed with PBS and incubated with blocking buffer 2 h at RT. The samples were incubated overnight at 4 °C with purified rabbit anti-elastase antibody (1:100); then they were rinsed twice with PBS-T and incubated with AlexaFluor 488-conjugated secondary antibody (1:500) for 2 h at RT. Finally, the specimens were rinsed twice with PBS-T and mounted with propidium iodide (PI)(25 μg/ml). Images were acquired with an ApoTome II microscope and recorded using the Axiovison 2.0 software (Carl Zeiss, Jena, Germany). For each condition, five randomly selected images were acquired and used for quantification of NET releasing cells. Times of exposition on each channel were kept constant. The images files were loaded as separate image stacks for each channel in Image J software. To quantify the traps, the brightness and contrast was first adjusted to every image to visualize each NET (composed of extracellular elastase and PI). Then binary images were generated and the threshold was subtracted. Subsequently automatic particle analysis was set to 20 pixels minimum size. The summarized result was considered as the area occupied by each liberated NET. The summarized result was considered the area occupied by the NET released. Data were expressed as percentages of NET-releasing cells in relation to the cells without LPS, as previously was reported^68^.

### Inhibition of NETs with rhTSG-6

Mouse neutrophils were stimulated for NETs induction and 4 × 10^4^ mouse neutrophils were seeded on 13 mm poly-*L*-lysine charged glass coverslips in 24-well-plates in RPMI; the plates were incubated for 20 min at 37 °C to allow adhesion of the cells. After the cell adhesion, serial dilutions of rhTSG6 were added to the neutrophils. The dilutions of rhTSG-6 were made from a stock solution of 1000 pg/ml using a dilution factor of 1:2, until at final concentration of 125 pg/ml. Then the cells were incubated for 90 min at 37 °C and 5% CO_2_ atmosphere. Subsequently, after finishing the period of incubation, the neutrophils were fixed, blocked and analyzed by immunofluorescence, as described before.

### Nitroblue Tetrazolium (NBT) assay

The NBT reduction assay is used to quantify reactive oxygen species like superoxide anion. A product of the reduction of NBT is the generation of blue–black formazan precipitates inside the cells. A stock solution of 10 mg diluted in 1 ml of deionized water was prepared immediately before use and was kept at 4 °C on the darkness; then a work solution of 0.3% of NBT was prepared. Similar to NET induction, mouse neutrophils (2 × 10^5^) were seeded on 13 mm poly-*L*-lysine charged glass coverslips in 24-well-plates in RPMI; the plates were incubated for 20 min at 37 °C to allow adhesion of the cells; subsequently, the cells were exposed or not to LPS in the presence or not of the different the conditioned media; afterwards, the work solution of NBT was added to the cells obtaining a final concentration of 0.1% of NBT then the cells were incubated for 30 min at 37 °C and 5% CO_2_ atmosphere. Subsequently, after finishing the period of incubation, the supernatants of each condition were removed and the cells were fixed with methanol for 5 min at RT. Afterwards, the fixed cells were rinsed again with 70% methanol and were air-dried in the darkness. Finally, the precipitates of formazan were solubilized in 260 μl containing 120 μl of 2 M KOH and 140 μL of DMSO. After homogenization, the suspension of each well was loaded into a well of 96-wells-plate to be read on a spectrophotometer (Multiskan Ascent, Thermo Labsystems, PHL, USA) at 630 nm. The O^2^- was determined as nanomoles (nM) of reduced NBT (NBTr) per well, considering an extinction factor of 0.1 OD at 630 nm representing 1.9 nM O^2^-^69^. The O^2^- production generated by the neutrophils without stimulus in fresh medium was considered the 100% of ROS production.

### Measurement of mitochondrial membrane potential (Δψ_m_)

The measurement of the mitochondrial membrane potential (Δψ_m_) indicates the functions of the mitochondria such as reactive oxygen species production among others. The inhibition of the Δψ_m_ is related to the decrease of the fluorescence of the lipophilic cationic probe DiOC6(3) in the cell. This assay was performed in suspension as follows: murine neutrophils (2 × 10^5^) were exposed to different stimuli during 15 min at 37 °C and 5% CO_2_ atmosphere. When incubation was finished, 30 nM of DiOC6(3) was added and the cells were incubated for additional 15 min at RT in the darkness. The excess of DiOC6(3) was rinsed with PBS and the cells were acquired immediately by flow cytometer^70^. The data are expressed as the percent of the Δψ_m_ loss (%Δψ_m_ loss).

### Statistical analysis

Data were collected and non-parametric Mann-Whitney *U* tests were performed, considering *p < 0.05 as statistically significant using the Prism 5 GraphPad software (La Jolla, California, USA).
